# Evaluation of Coagulants and Polymers for Optimizing Wastewater Treatment and Acid Oil Extraction in a Poultry Processing Plant

**DOI:** 10.3390/polym18091078

**Published:** 2026-04-29

**Authors:** Elisa Tschaen Schneider, Polyana Silverio Massariol, Viviane Martins de Deus, Caio Lucas Alhadas de Paula Velloso, Job Teixeira de Oliveira

**Affiliations:** 1 Department of Chemical Engineering, University of the Central East—Manguinhos, Serra 29173-087, ES, Brazil; elisa@oifrango.com.br (E.T.S.); polyanasilverio@ucl.br (P.S.M.); 2 Department of Food Technology, Federal University of Ouro Preto (UFOP), Ouro Preto 35400-000, MG, Brazil; vivianemartinsdd@gmail.com; 3 Department of Agricultural Engineering (DEA), Federal University of Vicosa (UFV), Vicosa 36570-900, MG, Brazil; caio.alhadas@ufv.br; 4 Department of Agronomy, Chapadão do Sul Campus (CPCS), UFMS—Federal University of Mato Grosso do Sul, Chapadão do Sul 79560-000, MS, Brazil

**Keywords:** ferric chloride, fatty acid oil, process optimization, poultry wastewater, dissolved air flotation, Monte Carlo simulation, polymers, sustainability

## Abstract

The treatment of oily wastewater represents a significant environmental challenge, requiring efficient separation technologies and waste valorization. This study evaluated different types of coagulants (ferric chloride 38% m/m, aluminum polychloride 18% m/m, aluminum sulfate 8% m/m, and ferrous sulfate 6% m/m) and anionic polymers (from six suppliers) for treating poultry slaughterhouse effluent, aiming to optimize both clarification and oil recovery from the floated sludge. Bench-scale jar tests (G = 300 s^−1^ and 30 s^−1^) were followed by full-scale validation in a dissolved air flotation unit (100 m^3^ h^−1^) at a poultry processing WWTP. Recovered oil was extracted by hot cooking (95 °C) and tridecanter centrifugation, and its quality (moisture, acidity, saponification index) was assessed. A techno-economic analysis, including simple/discounted payback, NPV, IRR, Monte Carlo simulation (10,000 iterations, Python), and deterministic sensitivity analysis, was performed. Ferric chloride (38% m/m) produced the best technical results: treated effluent turbidity < 30 NTU, oil yield of 360 L day^−1^ with moisture < 2% at the tridecanter outlet, and consistent sludge dewaterability (moisture 55–65%). Oil moisture increased dramatically (to >30%) after storage due to condensate contamination from an inefficient exhaust system, a critical operational flaw that must be corrected. No statistically significant effect of polymer type on oil recovery was observed, although high variability (CV > 50%) was noted during PAC tests. The simple payback period for ferric chloride was 60.7 months (discounted: 64.1 months), with a positive median NPV (USD 7925) under a 12% p.a. discount rate. Sensitivity analysis showed that the investment is most sensitive to oil price: a 20% drop in oil price leads to a negative NPV (−USD 21,727). Despite this risk, the project provides environmental compliance and waste-to-value benefits. The study demonstrates that ferric chloride enables effective oil extraction from poultry wastewater, but proper exhaust design is essential to maintain oil quality. Future work should focus on standardized test durations (≥72 h) and automated monitoring to reduce variability.

## 1. Introduction

The poultry processing industry has been growing exponentially in recent years. According to the 2024 report by the Brazilian Association of Animal Protein [1], 14.833 million tons of chicken meat were produced, with 65.35% destined for the domestic market and 34.65% for export, placing Brazil as the second largest producer in the world, behind only the United States of America [2]. The poultry processing industry has a high water consumption to meet all the regulatory requirements of the Ministry of Agriculture and Livestock; consequently, large volumes of industrial effluent are generated daily.

Large quantities of liquid and solid waste are generated daily in poultry processing plants due to the production process and all legal sanitary requirements. Ministry of Agriculture and Livestock (MAPA) Ordinance No. 210/1998 regulates the production process and installation, operation, and inspection requirements for poultry meat. To meet all MAPA requirements, the average water consumption can reach 20 L bird^−1^ [3]. This effluent is rich in fats and proteins, originating from the chicken meat and blood [4]. Typical raw poultry slaughterhouse wastewater contains oil and grease concentrations ranging from 1197 mg L^−1^ [5] to over 33,000 mg L^−1^ [6], with COD values between 1800 and 7200 mg L^−1^ [7,8]. Without adequate treatment, such effluents cause severe environmental impacts.

Conventional primary treatment relies on coagulation–flocculation followed by dissolved air flotation (DAF). Coagulation neutralizes the negative surface charges of colloidal particles, primarily through charge neutralization (using inorganic coagulants like aluminum or ferric salts) or sweep flocculation at higher dosages [9,10]. Subsequently, high-molecular-weight anionic polymers promote bridging and floc aggregation, enhancing solid–liquid separation [11]. The efficiency of this process depends on coagulant type and dosage, polymer properties (molecular weight, charge density, and chain architecture), pH, and mixing conditions.

Although numerous studies have evaluated coagulants for poultry wastewater treatment, most have focused on conventional parameters such as turbidity, COD, and suspended solid removal [5,7,12]. For example, Rodrigues [5] reported COD removal efficiencies of 60–80% using aluminum sulfate and ferric chloride, while Dallago et al. [8] achieved nitrification–denitrification in biological stages. However, the literature lacks a systematic comparison of how different coagulant–polymer combinations affect not only effluent clarification but also the subsequent recovery of lipids from the floated sludge—a critical step for waste valorization.

Specifically, the properties of the polymer (anionic vs. non-ionic, molecular weight, and charge density) directly influence floc structure, dewaterability, and the release of intracellular or emulsified oils during thermal or mechanical breaking. High-molecular-weight polymers may produce rubbery, cohesive flocs that retain oil, whereas lower-molecular-weight or different charge-density polymers can yield friable flocs that release lipids more readily during centrifugation. To date, no study has systematically varied polymer type (from multiple suppliers) while keeping the coagulant constant to isolate the polymer’s role in oil extractability from DAF sludge. Furthermore, existing research rarely validates bench-scale jar test results in full-scale DAF units operating at real-world flow rates (e.g., 100 m^3^·h^−1^), and even fewer integrate oil recovery via industrial tridecanter centrifugation with a rigorous financial feasibility analysis [12] that includes uncertainty [13] (Monte Carlo simulation).

Table 1 summarizes the available literature data on coagulant–polymer performance in poultry slaughterhouse wastewater treatment and oil recovery. Rodrigues [5] reported high removal efficiencies for oil and grease (97% with ferric sulfate) and turbidity (95–98%) using both inorganic and organic coagulants, but did not quantify the daily oil yield nor the polymer dosages applied. Moreover, that study focused on acidity of the recovered oil (7.57 mg KOH g^−1^ with ferric sulfate) without assessing the economic feasibility of oil production. Jiménez-Urpi et al. [6] provided a comprehensive review of slaughterhouse waste valorization via anaerobic digestion, but did not evaluate direct oil extraction by centrifugation after coagulation–flocculation. Consequently, no previous study has combined systematic screening of coagulants and polymers (with supplier-specific anionic polymers at defined dosages) with full-scale DAF validation at 100 m^3^ h^−1^, tridecanter-based oil recovery with quantified yield (360 L day^−1^) and quality (moisture < 2%), or Monte Carlo uncertainty analysis of financial returns. The present study fills these gaps by providing an integrated techno-economic framework that is directly replicable by the poultry industry.

The scarcity of studies that provide a quantified oil recovery yield (in L per day) along with full-scale coagulant and polymer dosages justifies the need for the present research. Thus, the research gaps addressed by this study are the lack of quantitative comparison of coagulants (ferric chloride, aluminum polychloride, aluminum sulfate, and ferrous sulfate) and polymers (varying supplier and molecular weight) on both DAF effluent quality and oil extraction yield, the absence of field-validation data under continuous operation, and the need for an economic model that incorporates market and operational variability.

To fill these gaps, this study advances current knowledge by pursuing the following specific objectives:

To evaluate, via bench-scale jar tests, the clarification efficiency and sludge characteristics (friability, plasticity) of different coagulant–polymer combinations using raw poultry slaughterhouse effluent.

To validate the most promising combinations in a full-scale DAF unit (100 m^3^·h^−1^) at a poultry processing WWTP, measuring turbidity (<30 NTU), sludge dewaterability, and oil production volume.

To quantify the quality of the recovered oil (moisture content, acidity, saponification index) after hot cooking (95 °C) and tridecanter centrifugation.

To compare the technical and financial performance of ferric chloride (38% m/m) and aluminum sulfate (8% m/m) in terms of Net Present Value (NPV), Internal Rate of Return (IRR), simple and discounted payback periods, and Monte Carlo uncertainty analysis (10,000 iterations).

By achieving these objectives, the study provides a replicable, integrated framework for converting floated sludge from an environmental liability into a marketable acid oil, while meeting stringent discharge limits (vegetable oils and animal fats < 50 mg L^−1^, pH 5–9, BOD removal > 60% according to Brazilian law 430/2011 [14]).

## 2. Materials and Methods

The study was conducted at the wastewater treatment plant (WWTP) of a poultry slaughterhouse located in the city of Marechal Floriano, Espírito Santo, at the coordinates 317,356.83 m E, 7,744,321.44 m S. The unit treats wastewater from the entire industrial poultry slaughtering process. The treatment has four main stages: screening, primary physical–chemical treatment, biological treatment (activated sludge), and secondary physical–chemical treatment.

The oil production process using the tridecanter started in December 2024, but to evaluate the floated sludge generated in the second stage of treatment, bench tests (jar tests), field tests, and technical and economic feasibility analysis were carried out. The centrifugal decanter and centrifugal tridecanter equipment use centrifugal force to separate the components of the mixture that have different densities; the decanter separates into two phases and the tridecanter into three phases. They achieve higher flow rates than the press, reaching up to 250 m^3^ h^−1^ [15]

### 2.1. Bench Test

The jar test procedure followed standard practices for coagulation–flocculation optimization [16]. Raw effluent samples were collected after the screening stage, homogenized, and used within 2 h. Tests were conducted using a six-paddle jar test apparatus (Model JT-6, Miltec, São Paulo, Brazil) with 1 L square jars at room temperature (25 ± 2 °C). Initial pH was measured with a benchtop pH meter (Hanna HI2221, Woonsocket, RI, USA).

For each test, 1 L of raw effluent was poured into a jar. The coagulation step consisted of rapid mixing at a mean velocity gradient G ≈ 300 s^−1^ (≈250 rpm, typical for this equipment geometry [16]) for 1 min. Coagulant (ferric chloride 38% m/m, aluminum polychloride 18% m/m, aluminum sulfate 8% m/m, or ferrous sulfate 6% m/m) was added immediately after starting rapid mixing, at dosages ranging from 100 to 500 ppm based on preliminary screening.

After rapid mixing, the speed was reduced to provide slow mixing at G ≈ 30 s^−1^ (≈35 rpm) for 15 min to promote flocculation. Anionic polymers (0.1% m/m solution, from six different suppliers) were added 1 min after the beginning of slow mixing, at dosages of 2000, 3000, or 5000 ppm. Polymer stock solutions were prepared fresh daily by dissolving the polymer powder in distilled water under gentle agitation for 1 h.

Following flocculation, mixing was stopped, and the flocs were allowed to settle for 20 min—a standard settling time for jar tests in slaughterhouse wastewater [5,16]. A 50-mL supernatant sample was then collected from 2 cm below the surface for turbidity measurement (Hach 2100Q turbidimeter, Loveland, CO, USA.). The remaining supernatant was filtered through two layers of Perfix^®^ cloth, Liaoning Province, China (orqualitative filter paper) to evaluate sludge floc characteristics (friability, plasticity, and fragmentation upon manual handling).

Coagulant dosages were optimized empirically by varying the coagulant concentration from 100 to 500 ppm in 50 ppm increments, while keeping the pH at the natural value of the effluent (pH 6.0–7.0). The optimal dosage was defined as the lowest concentration that produced clarified effluent turbidity < 30 NTU and flocs that settled within 20 min. For ferric chloride, the optimal dosage was 120 ppm; for aluminum polychloride, 100 ppm; for aluminum sulfate, 250 ppm; and for ferrous sulfate, 500 ppm. Higher dosages did not further improve turbidity and increased sludge production. This empirical approach, while not based on charge demand calculations, is standard industrial practice for coagulant selection [16].

When necessary, pH correction (using 1 M NaOH or 1 M H_2_SO_4_) was tested in subsequent runs. All jar test experiments were conducted in triplicate using fresh effluent samples collected on different days. The results are reported as mean ± standard deviation. Table 2 contains the data for the polymers.

Although preliminary visual assessments were made during jar tests (e.g., floc friability), all critical decisions (approval of coagulant/polymer, validation in the WWTP, oil extraction) were based on quantitative parameters: turbidity, sludge moisture, oil yield (L day^−1^), and oil moisture. The visual terms are only used to describe initial screening and do not affect the final conclusions.

### 2.2. Field Test (WWTP)

With the selection of the best-performing coagulants and polymers, the products were acquired for field testing at the WWTP.

For coagulant testing, the coagulant was placed in the chemical product tank. Then the hose and dosing pump needed to be cleaned internally with water to avoid cross-contamination. To start the test, the effluent flow rate to be dosed was calculated considering the test jar, using Equation (1).

(1) $$Qcoagulant=\frac{Djar\;test\times Vflotation}{1,000,000}$$

where we have:

Qcoagulant = theoretical coagulant dosing flow rate (L h^−1^)

Djar test = dosage performed in the test jar (ppm)

Vflotador = operating flow rate of effluent in the flotation unit (L h^−1^)

The polymer was prepared at 0.1% m m^−1^, and the theoretical flocculant dosage was calculated according to Equation (2).

(2) $$Qpolymer=\frac{Djar\;test\times Vflotation}{1,000,000}$$

where we have:

Qpolymer = theoretical polymer dosing flow rate (L h^−1^)

Djar teste = dosage performed in the test jar (ppm)

Vflotador = effluent operating flow rate in the flotation unit (L h^−1^)

The equipment used for the field test was the Poseidon model dissolved air flotation unit, manufactured by Fast [15], operating at 100 m^3^ h^−1^.

After defining the theoretical dosage, the dosing pumps were adjusted to apply the necessary theoretical flow rate. A sample was collected in the mechanical flocculator, and the chemical reaction was observed. If it occurs as per the jar test, the dosage should be maintained, and the result in the flotation unit should be awaited for at least 30 min. If the same result is not observed, the coagulant and polymer flow rates should be varied according to what is observed in the samples. For coagulation failure situations, the coagulant dosage should be adjusted, and for flocculation and clarification failure, the polymer dosage should be adjusted. Due to these adjustments, the actual flow rate in the equipment may differ from the theoretical flow rate of each product.

After adjusting the chemical dosages, the operation should be observed, and the pH and turbidity of the treated effluent should be analyzed using a benchtop pH meter and a benchtop turbidimeter. The sludge floc formed should be observed to see if it has a firm and consistent shape. The sludge consistency was quantitatively assessed by measuring its moisture content (after centrifugation) and its specific resistance to filtration (SRF, m kg^−1^). Crumbly sludge typically exhibited moisture of 55–65% and SRF < 1 × 10^12^ m kg^−1^, whereas rubbery sludge had moisture > 70% and SRF > 5 × 10^12^ m kg^−1^. The effluent at the outlet of the flotation unit must have a turbidity of less than 30 NTU throughout the process to meet the company’s environmental requirements.

Next, the treatment of the floated sludge began. This product was pumped into a 10,000 L heating tank, which was heated to 95 °C by direct and indirect heat injection, and was initially cooked for at least 40 min. At the end of the cooking time, the cooked sludge needed to be centrifuged in the tridecanter. The available tridecanter operates at 3000 rpm and has a capacity of up to 5 m^3^ h^−1^, but normal operation works with up to 2.8 m^3^ h^−1^. Subsequently, the centrifuged sludge was sent with a screw conveyor to the disc dryer, where the dried product should have a moisture content of less than 30%.

After the tridecanter began operating, the result of the separation of the 3 phases was observed: water, oil, and sludge. The water phase was collected and analyzed for turbidity (Hach 2100Q, NTU, Loveland, CO, USA) and oil and grease content (EPA method 1664, Washington, DC, USA). A whitish appearance corresponded to turbidity < 30 NTU and residual oil < 50 mg L^−1^, which were used as quantitative criteria. For the process to reach the efficiency desired by the company, the sludge phase must exhibit a moisture content between 55 and 65% and a particle size distribution where >90% of the dry solids pass through a 2 mm sieve (measured by dry sieving), while the oil phase must come out as a liquid, with a low water content (less than 2%), acidity less than 5% and a saponification index less than 450 mg g^−1^. Moisture and acidity of the recovered oil were analyzed in the company’s internal laboratory following the Brazilian Pharmacopoeia [17]. Saponification index analyses were then performed (in an external laboratory) to verify the quality of the oil produced.

All tests performed had a minimum duration of 48 h. After identifying the best results, it was necessary to operate for at least 1 week to validate the results. During each field test, effluent samples for turbidity and pH analysis were collected every 6 h. Oil production was measured daily. The reported values correspond to the average of at least three measurements taken during stable operation (coefficient of variation < 10%).

### 2.3. Analysis of Technical and Financial Feasibility

After conducting all field tests and validating the analysis results, the best field results were selected, and the financial aspects were evaluated. The prerequisite for the financial analysis was to be approved in the technical analysis, considering the standards defined by the company as satisfactory, namely, effluent turbidity at the flotation outlet of less than 30 NTU, oil with moisture of less than 2%, and oil yield greater than 250 L per day, which were defined in the previous step. Oil samples were collected in triplicate from the tridecanter outlet and from the storage tank. Each sample was analyzed in duplicate for moisture and acidity following the Pharmacopoeia methodology [17]. Results are expressed as mean ± standard deviation. In the financial analysis, the acquisition cost of the chemical product, the expected monthly consumption, and the product’s sales value were considered. The economic analysis considered only the incremental costs and revenues directly associated with the change in coagulant and the production of acid oil. The following costs were excluded because they are already incurred by the company regardless of the coagulant used and do not vary with the tested alternatives:

Labor: the same operators run the WWTP and tridecanter independently of the coagulant type; no additional staff were hired.

Electricity and steam: the DAF unit, tridecanter, and dryer operate continuously at fixed capacity (100 m^3^ h^−1^, 3000 rpm); their energy and steam consumption do not change when switching between coagulants.

Equipment depreciation: the tridecanter, dryer, and oil tank had already been purchased (investment of USD 226,415.09) and are used exclusively for oil recovery, not for treatment per se; their depreciation is considered in the payback calculation as initial investment, not as an operating cost.

Thus, only the costs of coagulants (ferric chloride vs. aluminum sulfate) and polymer, plus the revenue from oil sales, were included in the incremental cash flow. This approach isolates the financial impact of the coagulant switch and the oil valorization, which is the focus of the study.

### 2.4. Analytical Methods

All analyses were performed according to standard methods, as detailed below.

#### 2.4.1. pH and Turbidity

The pH of raw and treated effluent was measured using a benchtop pH meter (Hanna Instruments HI2221, Woonsocket, RI, USA), calibrated daily with standard buffer solutions (pH 4.0, 7.0 and 10.0). Turbidity was determined with a benchtop turbidimeter (Hach 2100Q, Loveland, CO, USA), following EPA Method 180.1. The instrument was calibrated with formazin standards (0, 20, 100, 800 NTU) before each measurement series.

#### 2.4.2. Chemical Oxygen Demand (COD)

COD was analyzed by the closed reflux colorimetric method (APHA Standard Method 5220 D, Washington, DC, USA). Samples were digested in a COD reactor (Hach DRB200, Loveland, CO, USA) at 150 °C for 2 h and measured at 620 nm using a spectrophotometer (Hach DR3900, Loveland, CO, USA). Results are expressed in mg O_2_ L^−1^.

#### 2.4.3. Biochemical Oxygen Demand (BOD)

BOD was determined by the 5-day incubation method (APHA Standard Method 5210 B, Washington, DC, USA). Dissolved oxygen was measured before and after incubation at 20 ± 1 °C using an oximeter (Hach HQ40d, Loveland, CO, USA). Nitrification was inhibited by adding 2-chloro-6-(trichloromethyl)pyridine (TCMP, Loveland, CO, USA).

#### 2.4.4. Oil and Grease (O&G)

Oil and grease were quantified by Soxhlet extraction with n-hexane (APHA Standard Method 5520 D, Washington, DC, USA). A 500 mL sample was acidified to pH < 2 with HCl and extracted with n-hexane. The solvent was evaporated, and the residue was gravimetrically determined.

#### 2.4.5. Total Solids

Total solids were determined by evaporating a 100 mL sample at 105 °C to constant weight (APHA Standard Method 2540 B, Washington, DC, USA).

#### 2.4.6. Sludge Moisture Content

Moisture of centrifuged sludge (from the tridecanter) was determined by drying approximately 10 g of sample at 105 °C for 24 h (gravimetric method, adapted from Brazilian Pharmacopoeia [17]).

#### 2.4.7. Oil Moisture Content

Oil moisture was measured using the Dean–Stark distillation method (ASTM D95, West Conshohocken, PA, USA) or, alternatively, by the Karl Fischer coulometric titration (Mettler Toledo C20, USA, Columbus, OH, USA), following the Brazilian Pharmacopoeia [17]. For routine analysis, the Dean–Stark method was used (100 mL of oil, refluxed with toluene for 2 h).

#### 2.4.8. Oil Acidity (Free Fatty Acids)

The acidity index (expressed as mg KOH g^−1^ or % oleic acid) was determined by titration according to the Brazilian Pharmacopoeia [17] and AOCS Official Method Cd 3d-63. Approximately 5 g of oil was dissolved in 50 mL of neutralized ethanol:diethyl ether (1:1) and titrated with 0.1 M KOH using phenolphthalein as indicator.

#### 2.4.9. Saponification Index

The saponification index (mg KOH g^−1^) was determined by refluxing 2 g of oil with 25 mL of 0.5 M ethanolic KOH for 1 h, followed by titration with 0.5 M HCl (AOCS Official Method Cd 3–25, Urbana, IL, USA).

### 2.5. Economic Modeling

For the economic feasibility analysis, the cost–benefit analysis methodology was used, based on consolidated literature references [18]. The main financial indicators calculated were the Net Present Value (NPV), the Internal Rate of Return (IRR), and the Investment Payback Period. All costs mentioned were analyzed using simple and compound payback methods. The acid oil in this study, in the financial market, is similar to free-acidity fatty acids, used as input in the biodiesel production process. In the quotations carried out in June 2025, proposals ranging from USD 0.57 to USD 1.08 were found in reputable companies in the sector that sells the products, such as Aboissa, MF Rural, and Abisa. This study considered an average price of USD 0.87 for calculation purposes and considered the investment in the tridecanter, dryer, and oil tank equipment to be USD 226,415.09.

The Net Present Value (NPV), which represents the present value of future net savings, was calculated using Equation (3):

(3) $$NPV=\sum_{j=0}^{n}\frac{{FC}_{j}}{{\left(1+i\right)}^{j}}$$

where n: project duration (10 years); j: annual period; FCj: incremental cash flow in year j; i: average attractive rate (MARR = 12% p.a.).

The Internal Rate of Return (IRR) was calculated as the discount rate that makes the NPV equal to zero (Equation (4)), representing the profitability of the project:

(4) $$IRR=\sum_{j=0}^{n}\frac{{FC}_{j}}{{\left(1+i\right)}^{j}}=0$$

The payback period was defined as the time required for the accumulated savings to equal the initial investment [19].

The discount rate of 12% p.a. reflects the Weighted Average Capital Cost (WACC), suitable for evaluating industrial infrastructure projects.

In addition to the simple payback period, the compound payback period (discounted payback) was calculated, which considers the time value of money, discounting the incremental cash flows by the discount rate (WACC of 12% p.a.). The compound payback period represents the time required for the accumulated present value of the cash flows to equal the initial investment. The equation used was:

(5) Composite Payback=t e j=1∑t(1+i)jFCj=0

where FCj is the incremental cash flow for the year, and j and i are the discount rates.

#### Uncertainty Analysis via Monte Carlo Simulation

Monte Carlo simulation represents a widely recognized probabilistic technique for evaluating the robustness of economic and engineering models in the face of multiple sources of uncertainty. In the context of industrial infrastructure projects, its application allows for the quantification of risks associated with key variables, such as operational costs, intervention effectiveness, and discount rates, providing more realistic and reliable views for decision-making [20].

To quantify the impact of market and operational uncertainties, a Monte Carlo simulation was performed using a custom Python script (version 3.10) with the library NumPy (v1.26). The simulation ran 10,000 iterations, each representing one possible year of operation. The following input variables were assigned probability distributions based on real operational data and market quotations (June 2025).

Oil price (USD L^−1^): triangular distribution with minimum = 0.64, most likely = 0.69, maximum = 0.87 (data from three Brazilian biodiesel feedstock traders: Aboissa, MF Rural, Abisa). The triangular distribution was chosen because only three price points were available. Daily oil production (L day^−1^): normal distribution with mean = 360 L day^−1^ and standard deviation = 15 L day^−1^, truncated to the interval (320, 400). The normality assumption was verified by the Shapiro–Wilk test (*p* > 0.05) using 30 consecutive days of stable operation. Daily ferric chloride consumption (L day^−1^): normal distribution with mean = 320 L day^−1^ and standard deviation = 12 L day^−1^. A positive correlation (ρ = 0.7) was imposed between ferric chloride consumption and oil production using the Cholesky decomposition method (NumPy), because higher effluent flow rates both require higher coagulant dosage and generate more oil. Polymer cost (USD year^−1^): fixed at 2944.12 (no variability, as polymer consumption was stable across tests). Ferric chloride price (USD L^−1^): fixed at 0.48 based on the supplier quote for 2025.

For each iteration, the Net Present Value (NPV) was calculated using the same discount rate (12% p.a.) and project horizon (10 years) as in the deterministic analysis. This methodology, as demonstrated by Pompigna and Mauro [20], offers a robust quantitative basis for decisions in high-uncertainty scenarios, aligning with best practices for evaluating infrastructure projects in industries.

### 2.6. Statistical Analysis

All experiments were performed in triplicate unless otherwise stated. For the jar test (Section 2.1), each coagulant–polymer combination was tested three times using freshly collected effluent samples; the results for turbidity and floc characteristics were averaged. For the field tests (Section 2.2), the WWTP operated under each coagulant–polymer condition for a minimum of 48 h (up to 96 h), and effluent samples were collected every 6 h during stable operation. Oil production (L day^−1^) was recorded daily, and the values reported correspond to the mean of at least three consecutive days of stable operation. For oil quality analyses (moisture, acidity), three independent samples were collected from the tridecanter outlet and the storage tank, and each was analyzed in duplicate.

Data are presented as mean ± standard deviation (SD). Statistical comparisons between coagulants (ferric chloride vs. aluminum sulfate) were performed using a two-tailed paired *t*-test (assuming unequal variances) with a significance level of α = 0.05, using the software R version 4.2.

## 3. Results

### 3.1. Raw Effluent Characterization and Bench Test

As requested by the company where the study was conducted, the initial tests maintained the 18% Aluminum Polychloride (PAC) coagulant and tested various types of anionic polymers. The reference for the jar test was to obtain sludge with a better appearance than the current dosage in terms of texture, in which the wastewater treatment plant used the anionic polymer 913 (supplier B).

The raw effluent from the poultry slaughterhouse was characterized over 12 months (January–December 2025) to assess its variability. Table 3 summarizes the monthly values and the overall mean ± standard deviation for pH, COD, BOD, total solids, oil and grease, and turbidity. The effluent exhibited high organic load (mean COD 2776 mg L^−1^, BOD 1510 mg L^−1^) and considerable oil and grease content (mean 191.5 mg L^−1^), with turbidity averaging 741 NTU. These values are within the typical range reported for poultry slaughterhouse wastewater [5,8]. The relatively high standard deviations reflect seasonal variations in production and slaughterhouse operations.

### 3.2. PAC Coagulant 18% m m^−1^—Dosage of 100 ppm

The first test was performed with polymer 8175 from supplier A, dosing 3000 ppm of the polymer. Good floc formation and good clarification were observed. The sludge’s appearance was as desired, fragmenting when handled. After the test, it was noticed that the sludge could disperse with friction in the hand, while the current polymer does not allow this to happen. The product passed the test.

The second test was performed with polymer 934 from supplier B, dosing 3000 ppm of polymer. The polymer previously used by the WWTP was Supplier B 913. Compared to 934, which has a higher molecular weight than 913, the formation of a finer floc was observed, but it did not have high plastic cohesion after removing excess water. However, its behavior in the flotation unit should be observed, since a very fine floc can cause an increase in the turbidity of the treated effluent. The product passed the jar test.

The third test was performed with the Ifloc 101 polymer from supplier C, with a dosage of 2000 ppm polymer. It was observed that there was good flake formation, and the clarified product was good. The flake, when handled, did not have a high plastic cohesion appearance. The product was approved.

The fourth test was performed with the poli-91 polymer from supplier D, with 2000 ppm polymer. It was observed that despite the fine flake, the clarified product was good. The flake, when handled, did not have a sticky appearance. The product was approved.

The fifth test was performed with the 3080 and 3010 polymers from supplier E. For both polymers, the dosage used was 5000 ppm. Despite the higher polymer dosage, good flake formation was observed, and it was not sticky; the 3080 polymer was approved. While with polymer 3010, it failed to flocculate correctly, and the clarified liquid remained cloudy; product 3010 failed the test.

The last test was with polymers 220, 280, and 290 from supplier F, all at a dosage of 5000 ppm. All polymers tested had low efficiency in floc formation and clarified liquid with high turbidity; the products failed the jar test.

Tests with Other Coagulants

Next, a jar test was performed with the other coagulants mentioned previously. The best dosage results found were ferric chloride 38% m m^−1^, 120 ppm, and 2000 ppm of polymer. Good flocculation and clarified liquid with low turbidity; the product was approved. Ferrous sulfate 6% m m^−1^, 500 ppm, and 3000 ppm of polymer. Good flocculation and slightly turbid clarified liquid; performance in the flotation unit will be evaluated in the field. Aluminum sulfate (8% m/m, 250 ppm) with 2000 ppm of 0.1% polymer solution. Good flocculation and clarified liquid with low turbidity; the product was approved.

### 3.3. Comparison of Results

After all bench tests, the data were compiled in Table 4. Four polymers were selected for field testing, which had satisfactory technical results and the lowest dosage. The dosage of PAC 18% coagulant was found to be constant in all tests. All data are reported as mean ± standard deviation (SD). Differences between coagulants were considered statistically significant when *p* < 0.05 (paired *t*-test). Due to the operational nature of the study, statistical inference was not performed; however, the low coefficients of variation (<15%) indicate good reproducibility.

During bench tests, data from laboratory analyses of the raw and treated effluent held by the company were also analyzed. Data from the period of November 2024 to May 2025 were analyzed in order to verify the effluent characterization profile. Table 5 presents the mean and standard deviation of the results of these analyses for the parameters of interest (BOD, COD, oils and fats, pH, ammoniacal nitrogen, and total solids).

The National Environmental Policy (PNMA) is regulated by Law No. 6938, from 31 August, Brazil [21]. The law establishes the objectives, purposes, and mechanisms for the preservation, improvement, and recovery of environmental quality, seeking to guarantee conditions for socioeconomic development, national security, and the protection of human dignity. Law 430/2011 [14] provides for the minimum conditions and standards for effluent discharge, with each state having the autonomy to create more stringent regulations, according to their respective environmental impact studies. The law regulates some established minimum discharge standards, such as pH between 5 and 9, mineral oils up to 20 mg L^−1^, vegetable oils and animal fats up to 50 mg L^−1^, minimum removal of 60% of BOD, and total ammoniacal nitrogen of 20 mg L^−1^.

After the start of tridecanter operation (December 2024), an increase in BOD and COD was observed in the raw effluent (Table 5). A plausible hypothesis for this increase is that the clarified liquid returning from the tridecanter to the equalization tank—which originates from the cooked sludge—contains solubilized organic matter. During the cooking step (95 °C, 40 min), some insoluble nutrients (e.g., proteins and lipids) may be hydrolyzed and released into the aqueous phase. This resolubilized material would then recirculate to the equalization tank, contributing to the higher organic load. However, no direct measurements of the tridecanter return liquid (COD, BOD, or soluble nitrogen) were performed to confirm this mechanism. Therefore, the explanation remains a hypothesis that should be tested in future studies by analyzing the return liquid before and after cooking.

The results of the theoretical and actual dosage of each product are presented in Table 6. The operating flow rate of the flotation unit remained at 100,000 L h^−1^ during all tests performed.

Oil production during the ferric chloride test (72 h) averaged 360 L day^−1^, with a standard deviation of 45 L day^−1^ (coefficient of variation, CV = 12.5%). However, production was not uniform: on two out of three days, output exceeded 380 L, while on one day it dropped to 310 L. More importantly, during the tests with PAC (Table 3), oil production varied from 0 to 120 L per test period, with several days of zero production. This high variability (CV > 50%) was associated with operational instability of the DAF unit (fluctuating raw effluent flow and coagulant dosing). For the ferric chloride trials, after optimizing the coagulant dosage, the coefficient of variation decreased to <15%, indicating more stable oil recovery.

During field tests, a higher actual polymer consumption was observed with the Poli-91 product from Supplier D, as shown in Table 4. Regarding oil production and sludge drying, low and inconsistent results were observed, with some days showing production and others not. The oil showed improved viscosity upon visual inspection, but presented high moisture content, reaching up to 40%. Oil production values for the four anionic polymers (8175, 934, Ifloc 101, Poli-91) were 40, 120, 100, and 30 L per test period, respectively (Table 3). The mean production was 72.5 L (standard deviation = 41.5 L, coefficient of variation = 57%). This high variability overlaps with the day-to-day fluctuations in raw effluent composition (e.g., oil and grease ranged from 39 to 546 mg L^−1^, Table 1). Given the limited number of replicates (one test per polymer) and the absence of formal statistical testing, we refrain from claiming a statistically significant absence of effect. Instead, we conclude that, under the tested conditions, polymer type did not appear to be a dominant factor for oil recovery when using PAC 18% as coagulant. Floc friability, measured as the percentage of floc mass passing through a 2 mm sieve after gentle compression, was >75% for all polymers that passed the jar test. Polymer 934 (very high molecular weight, medium charge density) showed the highest friability (82%), which correlates with the ease of oil release during subsequent centrifugation (oil yield 120 L per test period). Polymers with lower molecular weight (e.g., 3010) produced friability < 30% and did not form stable flocs. In contrast, polymers with lower molecular weight (e.g., CHEMIFLOC 3010-X, high molecular weight) resulted in finer flocs and slightly higher turbidity in the treated effluent, but did not negatively affect oil extraction yield. These observations are consistent with the understanding that very high-molecular-weight anionic polymers promote bridging flocculation, while charge density influences floc strength and dewaterability [9,10]. Following this test, tests were initiated in the flotation unit with other coagulants: ferric chloride, ferrous sulfate, and iron-free aluminum sulfate. Table 7 presents the test results.

It should be noted that the field tests were conducted with different durations (6 h for ferrous sulfate, 24 h for aluminum sulfate, and 72 h for ferric chloride) due to operational constraints (immediate failure of ferrous sulfate, limited product availability for aluminum sulfate). Despite this limitation, the conclusions are robust because ferrous sulfate failed within the first 6 h (turbidity > 100 NTU, no floc formation), and extending the test would not reverse this failure; aluminum sulfate achieved acceptable effluent quality within 24 h, but its higher cost and lower oil yield compared to ferric chloride were already evident; and ferric chloride maintained stable performance over 72 h, confirming its suitability.

The 6% m m^−1^ ferrous sulfate coagulant was tested for 6 h at the wastewater treatment plant (WWTP), and the test was suspended due to unsatisfactory results regarding turbidity at the flotation outlet of greater than 100 NTU and the lack of floc formation after chemical dosing. Therefore, the product failed the field test. The 38% m m^−1^ ferric chloride and 8% m m^−1^ aluminum sulfate coagulants performed well in the tests, delivering turbidity of less than 30 NTU at the flotation outlet and producing a satisfactory volume of oil. The 8% m m^−1^ aluminum sulfate test was carried out in only one day due to the amount of product available for testing. The company showed interest in the product and will purchase a larger quantity of the product to test in the future.

After the tests were completed, the company opted to adjust the operation to continue using the 38% m m^−1^ ferric chloride coagulant. Therefore, it was possible to better evaluate the use of ferric chloride in the treatment, which delivered satisfactory results. Samples were collected from oils produced with 38% m m^−1^ ferric chloride coagulant and 8% m m^−1^ aluminum sulfate coagulant. Analysis of the results showed significant variation in moisture and acidity analyses, following the Pharmacopoeia methodology [17]. A sample was also collected from the oil storage tank after 30 days of operation, from 5 May to 26 May 2025. The results were compiled and are presented in Table 8.

### 3.4. Technical and Financial Feasibility Analysis

The tests selected for the financial feasibility analysis were those that presented effluent turbidity at the flotation outlet of less than 30 NTU, oil with moisture of less than 2%, and oil yield greater than 250 L per day, as defined in the previous step. The considered selling price of the oil was USD 0.87 kg^−1^, and the cost of the coagulants was USD 0.34 kg^−1^ for ferric chloride and USD 0.45 kg^−1^ for aluminum sulfate. Table 9 presents the results of chemical consumption and oil production considering a 24 h operation for 21 days a month. The fluctuation in the values of the chemical products and the oil throughout the months was not considered. Both coagulants, ferric chloride 38% m m^−1^ and aluminum sulfate 8% m m^−1^, were from Supplier D and polymer Supplier B 913. An oil density of 0.800 kg L^−1^ and a density of 1.4 kg L^−1^ were considered for both coagulants.

The 8% m m^−1^ aluminum sulfate coagulant proved to be financially unviable because the value obtained from the sale of oil was lower than the operating costs. In addition, it has a cost 32% higher than the 38% m m^−1^ ferric chloride coagulant.

**Figure 1:**
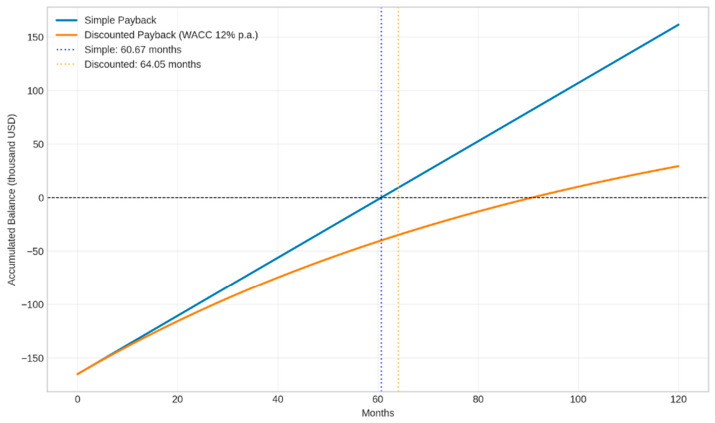
Simple payback analysis and discounted payback analysis using ferric chloride as a coagulant. Source: the authors (2026).

The comparison of cost and income generation results for the evaluated coagulants can be seen in Figure 1.

The company understands that it is advantageous to keep the process operational, given that the investment has already been made and the process now generates disposable income from the sale of the oil. Considering this outcome, the process is deemed viable since it eliminates an existing company cost, as the main objective of purchasing the equipment is to reduce operational costs and meet environmental compliance requirements.

### 3.5. Uncertainty Analysis

To quantify the variability of this benefit, a Monte Carlo simulation with 10,000 iterations was performed, based on real operational data and the following assumptions:

- Oil price: minimum of USD 0.64 L^−1^, most likely USD 0.69 L^−1^ and a maximum of USD 0.87 L, reflecting the prices actually practiced for oil with moisture < 2% and controlled acidity.
- Daily oil production: normal distribution with an average of 360 L day^−1^ and a standard deviation of 15 L day^−1^, within the range of (320, 400) L day^−1^—consistent with the operational stability achieved after optimization.
- Daily consumption of ferric chloride: normal distribution with a mean of 320 L day^−1^ and a standard deviation of 12 L day^−1^, positively correlated with production (ρ = 0.7), since higher flow rates require a higher dosage. Cost of ferric chloride: fixed at USD 0.48 L^−1^, according to the applied market price. Cost of the polymer: fixed at USD 2944.12 year^−1^.

**Figure 2:**
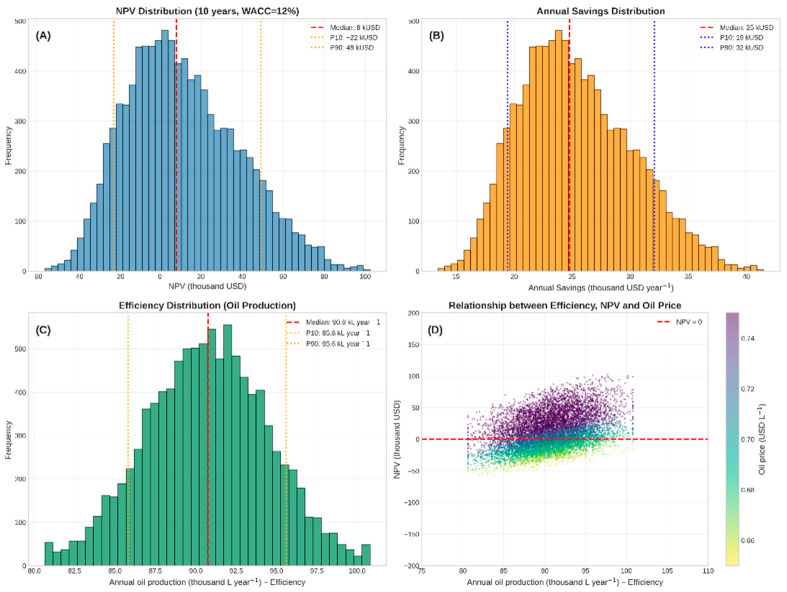
(**A**) Frequency distribution of the Net Present Value (NPV) obtained in the Monte Carlo simulation (10,000 iterations). (**B**) Distribution of annual savings. (**C**) Distribution of oil production efficiency. (**D**) Relationship between annual oil production efficiency and NPV, colored by the price of oil (USD L^−1^). Each point represents a simulation. The dashed red line indicates NPV = 0. The color scale represents the price of oil (USD L^−1^). Source: the authors (2026).

Figure 2 shows the frequency distribution of the Net Present Value (NPV) obtained in the Monte Carlo Simulation (10,000 iterations), the distribution of annual savings, the distribution of effectiveness, and the effectiveness ratio.

**Figure 3:**
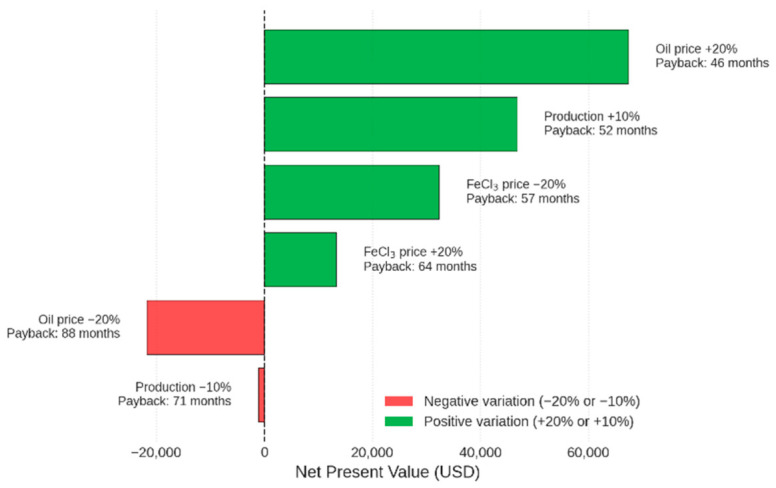
Deterministic sensitivity analysis (NPV variation). Source: the authors (2026).

To complement the Monte Carlo simulation, a deterministic sensitivity analysis was performed by varying one parameter at a time while keeping others at their base values (Figure 3). The following scenarios were tested: oil price ± 20% (USD 0.70–1.04 L^−1^), ferric chloride price ± 20% (USD 0.27–0.41 L^−1^), and daily oil production ± 10% (324–396 L day^−1^). Figure 1 presents the results as a tornado diagram, showing the impact of each variation on the Net Present Value (NPV) and simple payback period. The project is most sensitive to oil price: a 20% reduction would make the NPV negative (−USD 21,727) and extend the payback to 88.4 months. A 10% drop in oil production also leads to a slightly negative NPV (−USD 1107). In contrast, a 20% increase in coagulant cost reduces NPV by only 42% (to USD 13,312) and increases payback to 63.7 months. These findings indicate that the investment is robust under most realistic scenarios, but oil price volatility poses the greatest risk.

## 4. Discussion

### 4.1. Comparison of Results

The slaughterhouse effluent has a high fat content. Rodrigues [5] carried out the characterization of the effluent in his study and found a result of 1197.50 mg L^−1^ of oils and greases in the effluent from a poultry slaughterhouse. The study by Dallago et al. [8] in the characterization of poultry slaughterhouse effluent obtained a COD value of 7208.63 mg L^−1^ and a pH of 7.64. In another study analyzing waste from a slaughterhouse effluent treatment plant, a pH of 4.2 and a concentration of 33,000 mg L^−1^ were found [6]. The different results found in the research show that there are variations in the processes that can affect the organic load of the raw effluent, and can be verified by the considerable standard deviation results of each parameter over time.

The management of wastewater treatment plants (WWTPs) has great challenges, given the potential environmental impact that can be caused if serious non-conformities occur. To meet legal requirements, companies resort to professionals in the chemical and environmental field to design and manage the routines of operations. The ABNT ISO 14001 standard presents a model of an Environmental Management System, promoting a structure for environmental protection and responses to changes that may occur [11].

### 4.2. Field Test (WWTP)

An increase in moisture content was identified in the oil after it was transferred to the storage tank. Analysis of the company’s installation revealed that the tridecanter’s exhaust system was inefficient, promoting condensate formation throughout the network. Consequently, 3000 L of water were drained from the storage tank, and a sample was collected for analysis; however, the results had not yet been issued by the laboratory at the time of completion of this study. Such high moisture severely degrades oil quality: it promotes hydrolysis of triglycerides, increases free fatty acids during storage, and makes the oil unsuitable for biodiesel production without costly drying steps (commercial specifications typically require moisture < 0.5%). The company was contacted regarding the need to adapt the exhaust system to ensure that the oil is not contaminated with condensate. The company stated that it would adjust the structure and the necessary equipment. However, it was observed that even with a high concentration of moisture in the stored oil, there was a decrease in the oil’s acidity. Nunes et al. [22] studied the reduction in acidity in macauba pulp oil and observed a decrease in acidity after heating the oil to 180 °C. The present study does not aim to delve into the quality of the oil; other methods of neutralizing the acidity of oils are widely studied, such as washing with an alkaline solution, such as potassium hydroxide or sodium hydroxide, among others [23].

The large variability in oil production observed during the initial tests (PAC coagulant) can be attributed to several operational factors: fluctuations in raw effluent flow rate (80–120 m^3^ h^−1^) affecting coagulant residence time, inconsistent polymer dispersion due to manual preparation of stock solutions, or variations in the oil content of the incoming wastewater. Days with zero production typically occurred when the DAF unit experienced short-circuiting or when the sludge blanket was not properly discharged. After switching to ferric chloride and optimizing the dosage, the process became more robust: the coefficient of variation dropped from >50% to <15%. This improvement is likely due to the higher density and faster settling of ferric chloride flocs, which reduced sensitivity to hydraulic fluctuations. Nevertheless, occasional zero-production days still occurred (e.g., during maintenance shutdowns or when raw effluent O&G fell below 100 mg L^−1^). Future work should focus on online monitoring of oil content and automated coagulant dosing to further stabilize production.

The performance differences among the anionic polymers can be attributed to their molecular weight and charge density, although complete manufacturer data were not available for all products. Polymer 934 (FLONEX™ 934 SH), which has a very high molecular weight and medium charge density, produced flocs with good friability (estimated >80% disintegration upon gentle compression). Very high-molecular-weight anionic polymers promoted bridging flocculation, creating larger flocs that settled faster and released more interstitial water during compression [9,10]. In contrast, polymers with lower molecular weight (e.g., CHEMIFLOC 3010-X, high molecular weight, unspecified charge density) resulted in finer flocs and higher effluent turbidity, likely due to insufficient bridging and higher residual colloidal charge. Poli-91 (supplier D), whose ionic character was not explicitly stated, produced only moderate flocculation. Polymers that failed (e.g., 3010, 220, 280, 290) either had insufficient charge density to neutralize the colloidal particles or had molecular weights too low to form stable bridges. This interpretation is consistent with classical coagulation–flocculation theory, where anionic polyacrylamides of very high molecular weight (>15 MDa) are most effective for solid–liquid separation in DAF systems treating oily wastewater [10,11].

No measurements of zeta potential or surface charge were performed. Such analyses would help to understand the coagulation mechanism (charge neutralization vs. sweep flocculation) and to optimize coagulant dosage more precisely. However, the objective of this industrial study was practical: to identify a coagulant–polymer combination that meets discharge limits and enables oil recovery, not to characterize the colloidal chemistry in depth. Second, floc size and floc strength were not measured quantitatively. The jar test evaluation relied on a qualitative friability test (manual compression) and turbidity as a proxy for flocculation efficiency.

### 4.3. Financial Feasibility Analysis

The inflection point where the cash flow exceeds the investment balance, called the simple payback, was 60.67 months, while the discounted payback was 64.05 months. The small difference observed (about a 5.6% increase) indicates that the operating cash flow generated by the process (revenue from the sale of acid oil less incremental costs with coagulant and polymer) is relatively stable over the analyzed horizon. This stability mitigates the effect of the discount rate on the capital recovery time. Oliveira et al. [12] found, in a feasibility study with effluent treatment, a simple payback of 104.40 months (8.7 years), showing that the payback value found in this study demonstrates attractiveness.

The positive median NPV (USD 7924.93) indicates that, in the most likely scenario, the project adds economic value over 10 years, considering a MARR of 12% per year. The 80% confidence interval (P10 to P90) ranges from a small loss (USD −22,264.15) to a significant gain (USD 49,245.28), reflecting the project’s sensitivity to market and operational fluctuations. The probability of a positive NPV is greater than 60% (estimated by the position of the median and P10), which, for a project with an environmental and strategic focus, is an acceptable result and aligned with the company’s expectations. Investments in technologies improve production quality, increase income, and generate jobs [24,25].

The discounted payback (not shown in the graph, but calculated internally) is around 6 to 7 years on average, consistent with the simple payback of 60.67 months (≈5.05 years) reported in the deterministic study. The difference is due to the incorporation of uncertainties and the cost of capital.

The decision to invest in new equipment in the acid oil extraction system cannot be evaluated solely by traditional financial metrics. Several intangible and strategic gains were achieved, which fully justify the investment and should be considered in the overall analysis. In any business, including broiler chicken farming, profit is the objective to be achieved [26]. To obtain the highest possible profit in a business, particularly in the poultry sector, it is essential to consider many factors; among them, investors must be able to analyze the business’s revenues and expenses to determine the profitability of the poultry operation [27]. Furthermore, these same authors highlight that, as part of the evaluation, it is also necessary to analyze the viability of the business and the level of performance, since poultry farming is a promising business for the future.

The implementation of the system allowed the company to fully meet the requirements of environmental legislation (and state regulations) [14], especially regarding the discharge of effluents with controlled levels of oils and greases. Failure to comply would imply fines, operational restrictions, and damage to the institutional image. The proper disposal of floated sludge, with oil extraction and reduction of moisture to <30%, enables burning in a boiler or environmentally safe final disposal, avoiding environmental liabilities. The pressing issue of water scarcity has far-reaching consequences for global sustainability, with implications for the future of humanity [28,29]. A widely accepted strategy to address this water challenge is wastewater treatment, a concept that has become increasingly prevalent in various regions of the world [30,31].

Reducing costs associated with wastewater treatment and waste disposal (sludge) generates operational savings that, while not directly accounted for in the incremental cash flow, contribute to the overall efficiency of the wastewater treatment plant. The sale of acid oil diversifies the company’s revenue, reducing dependence on core activities and creating a new source of income in an expanding market (biodiesel, animal nutrition).

The treatment of oily wastewater represents a significant environmental challenge, creating a demand for advanced separation technologies [32]. Due to this factor, the operation of the system (flotation unit, tridecanter, dryer) required the hiring and training of new employees, generating direct and indirect jobs. Training the team in advanced wastewater treatment and oil extraction techniques adds value to the company’s human capital.

The extracted fatty acid oil has become part of the list of marketable byproducts, opening opportunities in segments such as the biodiesel industry, soap manufacturing, and animal feed, among others. The company may, in the future, explore refining or transesterification of the oil to add even more value. Current and emerging practices for the treatment and valorization of slaughterhouse waste, focusing on transforming waste into high value-added products, promote sustainability in the meat industry [6].

The initiative to recover oil from effluents, transforming waste into a product, strengthens the company’s image as environmentally responsible and aligned with the principles of the circular economy. This differential can be exploited in marketing campaigns, sustainability reports, and in communication with clients and investors who value ESG (Environmental, Social, and Governance) practices.

The environmental certifications or seals obtained from this process can open doors to more demanding markets and improve competitiveness. However, it is important and recommended that the government adopt an approach in policy formulation and in offering lines of credit to assist in the return on investment of actors in the broiler chicken value chain in the poultry industry [33].

A limitation of this study is that the field tests for different coagulants were not conducted over identical time periods. Ferrous sulfate was discontinued after 6 h because the effluent quality (turbidity > 100 NTU) was far below the required standard (<30 NTU) and no floc formation was observed; continuing the test would not have improved the outcome, as the coagulant was clearly ineffective under the tested conditions. Aluminum sulfate was tested for only 24 h because of limited product availability; however, even within this short period, its performance was inferior to ferric chloride in terms of oil yield (250 L day^−1^ vs. 360 L day^−1^) and coagulant cost (32% higher). Ferric chloride was tested for 72 h, providing a more robust dataset, but its superiority over aluminum sulfate was already apparent within the first 24 h. Therefore, while the different test durations introduce some heterogeneity, the main conclusions—that ferric chloride is the most effective and economically viable coagulant—are not undermined.

## 5. Conclusions

Tests demonstrated that there was no relationship between oil extraction and polymer variation using the PAC coagulant 18% m m^−1^. Replacing it with ferric chloride 38% m m^−1^ resulted in improved technical and economic performance in the wastewater treatment system. During bench tests and full-scale tests, the production of a more stable sludge was verified, and it was possible to extract oil with low moisture content, good quality, and a volume that allows for a financial return for the company. Tests with ferrous sulfate 6% m m^−1^ and aluminum sulfate 8% m m^−1^ proved to be inefficient and costly for the company, failing to achieve technical and financial viability.

The calculated simple payback period was 60.67 months, and it is concluded that the process meets the company’s technical requirements, due to the existing structure and the potential for continuous return from oil sales.

Therefore, it is necessary for the company to adapt the oil tank exhaust system to prevent the formation of condensable liquids inside the tank and pipes. Finally, it is recommended to research methods to improve the quality of the oil produced, whether through a chemical process in the flotation unit or by adding another treatment step, such as neutralization and transesterification. Different methods of burning this material and the available calorific potential for energy efficiency can also be studied.

The Monte Carlo simulation demonstrates that, under uncertainty, the investment presents positive financial viability in the median, with controlled risk (negative P10, but with low probability). However, the true value of the project transcends traditional economic indicators. The environmental, legal, social, and image gains position the company as a benchmark in sustainable waste management and create long-term competitive advantages.

It is therefore recommended that the feasibility analysis consider a multi-criteria approach, weighing non-financial benefits as an essential part of the decision. The company is already reaping the benefits of this investment, with stable operation, consistent oil production, and full environmental compliance, which validates the choice made and encourages the replication of the model in other industrial units.

## Figures and Tables

**Table 1:** Comparison of coagulants, polymers, treatment efficiency, and oil recovery in poultry slaughterhouse wastewater treatment.

Reference	Coagulant	Polymer (Type/Dosage)	Oil & Grease/Turbidity Removal	Recovered Oil Yield	Scale
Rodrigues [5] (2023)	Ferric sulfate	Not specified	O&G: 97%	Acidity: 7.57 mg KOH g^−1^ (no yield in L day^−1^)	Full-scale
			Turbidity: 95%		
Rodrigues [5] (2023)	Magnofloc LT7990 (organic)	Not specified	O&G: 91%	Acidity: 5.75 mg KOH g^−1^	Full-scale
			Turbidity: 98%		
Jiménez-Urpi et al. [6] (2025)	Review—various	Review—various	Compiled data (non-primary)	Not reported	Review
This study	FeCl_3_ 38% (120 ppm)	Anionic (2000–5000 ppm)	Turbidity < 30 NTU; O&G < 50 mg L^−1^	360 L day^−1^ (moisture < 2%, acidity ~23.8%)	Full-scale (100 m^3^ h^−1^)

**Table 2:** Characteristics of the anionic polymers tested in the jar test and field trials (according to manufacturer technical data sheets).

Polymer Code	Supplier	Ionic Type	Charge Density	Molecular Weight	Chemical Composition	Physical Form
8175	A	Anionic	Not specified	Not specified	Polyacrylamide	Granular
934 (FLONEX™ 934 SH, São Paulo, Brazil))	B (SNF)	Anionic	Medium	Very high	Polyacrylamide	White granular
Ifloc 101	C	Anionic	Not specified	Not specified	Polyacrylamide	Granular
Poli-91 (VS-POLI91, Vitória (ES) Brazil)	D	Not specified	–	High	Polyacrylamide	White granular
3080 (CHEMIFLOC 3080-X, São Paulo, Brazil))	E	Anionic	Not specified	Very high	Polyacrylamide	White granular

Note: For polymers where charge density or molecular weight were not provided by the manufacturer, the information is indicated as “not specified”. All polymers were prepared as 0.1% (m/m) aqueous solutions. The anionic nature was confirmed by the supplier documentation for all tested products, except for VS-POLI91 (supplier D), where the ionic character was not explicitly stated but is presumed to be anionic based on typical flocculant use in DAF systems for poultry wastewater.

**Table 3:** Physicochemical characterization of the raw poultry slaughterhouse effluent (monthly data from January to December 2025).

Month	pH	COD (mg O_2_ L^−1^)	BOD (mg O_2_ L^−1^)	Total Solids (mg L^−1^)	Oil & Grease (mg L^−1^)	Turbidity (NTU)
January	5.89	3024	1695	1786	39.4	718
February	6	3447	2048	1396	154.6	761
March	6.34	2839	1500	1562	202.8	730
April	6.21	4007	2456	1672	156	801
May	5.81	3071	2642	942	91.4	734
June	6.12	3164	1200	1612	138.4	732
July	6.2	3125	1665	1534	59.6	765
August	7.01	1175	563	1330	128.6	772
September	6.7	2553	1277	1432	220.5	741
October	6.54	1198	594	1772	275	690
November	6.17	3594	1795	2555	546.4	754
December	6.18	1109	668	1668	185.8	702
Mean ± SD	6.28 ± 0.35	2776 ± 1042	1510 ± 678	1605 ± 410	191.5 ± 139.4	741 ± 30

Note: Total solids represent the sum of suspended and dissolved solids. COD: chemical oxygen demand; BOD: 5-day biochemical oxygen demand; SD: standard deviation (n = 12 monthly samples).

**Table 4:** Results of polymer tests.

Anionic Polymer	Coagulant Used	Coagulant Dosage	Polymer Dosage 0.1%	Final Conclusion	Selected for Field Testing?
8175	PAC 18% m m^−1^	100 ppm	3000 ppm	Approved	Yes
934	PAC 18% m m^−1^	100 ppm	3000 ppm	Approved	Yes
Ifloc 101	PAC 18% m m^−1^	100 ppm	2000 ppm	Approved	Yes
Poli-91	PAC 18% m m^−1^	100 ppm	2000 ppm	Approved	Yes
3080	PAC 18% m m^−1^	100 ppm	5000 ppm	Approved	No
3010	PAC 18% m m^−1^	100 ppm	5000 ppm	Failed	No
220/280/290	PAC 18% m m^−1^	100 ppm	3000 ppm	Failed	No
913	Ferric chloride 38% m m^−1^	120 ppm	2000 ppm	Approved	Yes
913	Ferrous sulfate 6% m m^−1^	500 ppm	3000 ppm	Approved	Yes
913	Aluminum sulfate 8% m m^−1^	250 ppm	2000 ppm	Approved	Yes

Polymer dosages refer to the volume of 0.1% (m/m) stock solution added per liter of effluent. The corresponding active polymer concentrations are 2–5 ppm. Source: the authors (2026).

**Table 5:** Results of the WWTP analyses, raw effluent.

Collection Date	Ph	BOD (mg L^−1^)	COD (mg L)	Vegetable Oils and Animal Fats (mg L^−1^)	Ammoniacal Nitrogen (N-mg L^−1^)	Total Solids (mg L^−1^)
12 November 2024	6.00	1431.0	3536.0	54.0	24.0	1249.0
10 December 2024	6.29	831.0	1305.0	177.4	21.4	1306.0
14 January 2025	5.89	1695.0	3024.0	39.4	14.0	1786.0
11 February 2025	6.00	2048.0	3447.0	154.6	37.2	1396.0
11 March 2025	6.34	1500.0	2839.0	202.8	46.8	1562.0
8 April 2025	6.21	2455.9	4007.0	156.0	39.8	1672.0
15 May 2025	5.81	2642.0	3071.0	91.4	16.4	942.0
Average	6.08	1800.4	3032.7	125.1	28.5	1416.1
Standard deviation	0.20	629.40	856.2	63.4	12.7	285.4

Source: the authors (2026).

**Table 6:** Theoretical and actual dosage of PAC 18% m m^−1^ in the WWTP flotation unit.

Supplier	Anionic Polymer	Theoretical PAC Dosage: 18% m m^−1^ (L h^−1^)	Theoretical Polymer Dosage: 0.1%. (L h^−1^)	PAC Dosage 18% m m^−1^ Actual (L h^−1^)	Actual Polymer Dosage (L h^−1^)	Oil Production (L)	Test Period
Supplier A	8175	10	300	7.8	120	40	48 h
Supplier B	934	10	300	8.1	120	520	72 h
Supplier C	Ifloc 101	10	200	12.5	110	0	96 h
Supplier D	Poli-91	10	200	9.6	300	0	48 h

Source: the authors (2026).

**Table 7:** Theoretical and actual dosage of coagulants in the flotation unit.

Supplier	Coagulant	Theoretical Coagulant Dosage (L h^−1^)	Theoretical Polymer Dosage: 0.1%. (L h^−1^)	Actual Coagulant Dosage (L h^−1^)	Actual Polymer Dosage (L h^−1^)	Oil Production (L)	Test Period
Supplier D	Ferric chloride 38% m m^−1^	12	200	15	120	1080	72 h
Supplier D	Ferrous sulfate 6% m m^−1^	50	300	80	500	0	6 h
Supplier D	Aluminum sulfate 8% m m^−1^	25	200	20	120	250	24 h

Source: the authors (2026).

**Table 8:** Results of the analyses of the produced oil.

Sample	Coagulant Used	Collection Point	Moisture (%)	Oleic Acid (g 100 mL^−1^)
01	Ferric chloride 38% m m^−1^	Tridecanter outlet	1.20	23.90
02	Ferric chloride 38% m m^−1^	Tridecanter outlet	0.68	23.80
03	Ferric chloride 38% m m^−1^	Tridecanter outlet	1.68	n/a
04	Aluminum sulfate 8% m m^−1^	Tridecanter outlet	4.87	n/a
05	Aluminum sulfate 8% m m^−1^	Tridecanter outlet	2.34	n/a
06	Ferric chloride 38% m m^−1^	Storage tank	30.43	11.30

Source: the authors (2026). Legend: n/a = not applicable.

**Table 9:** Simulation of chemical consumption and oil production in the operation of the wastewater treatment plant.

Coagulant	Aluminum Sulfate 8% m m^−1^			Ferric Chloride 38% m m^−1^		
Duration	Coagulant Consumption (L)	Polymer Consumption (kg)	Oil Production (L)	Coagulant Consumption (L)	Polymer Consumption (kg)	Oil Production (L)
1 day	480	2.88	250	360	2.88	360
21 days	10,080	60.48	5250	7560	60.48	7560
Unit price	USD 0.45	USD 4.06	USD 0.87	USD 0.34	USD 4.06	USD 0.87
Total cost/revenue	USD 4526.49	USD 245.34	USD 783.02	USD 2567.55	USD 245.34	USD 6561.51

Source: the authors (2026).

## Data Availability

The original contributions presented in the study are included in the article; further inquiries can be directed to the corresponding authors.
